# Theta Rhythms Coordinate Hippocampal–Prefrontal Interactions in a Spatial Memory Task

**DOI:** 10.1371/journal.pbio.0030402

**Published:** 2005-11-15

**Authors:** Matthew W Jones, Matthew A Wilson

**Affiliations:** **1**The Picower Institute for Learning and Memory, Department of Brain and Cognitive Sciences, RIKEN-MIT Neuroscience Research Center, Massachusetts Institute of Technology, Cambridge, Massachusetts, United States of America; University of EdinburghUnited Kingdom

## Abstract

Decision-making requires the coordinated activity of diverse brain structures. For example, in maze-based tasks, the prefrontal cortex must integrate spatial information encoded in the hippocampus with mnemonic information concerning route and task rules in order to direct behavior appropriately. Using simultaneous tetrode recordings from CA1 of the rat hippocampus and medial prefrontal cortex, we show that correlated firing in the two structures is selectively enhanced during behavior that recruits spatial working memory, allowing the integration of hippocampal spatial information into a broader, decision-making network. The increased correlations are paralleled by enhanced coupling of the two structures in the 4- to 12-Hz theta-frequency range. Thus the coordination of theta rhythms may constitute a general mechanism through which the relative timing of disparate neural activities can be controlled, allowing specialized brain structures to both encode information independently and to interact selectively according to current behavioral demands.

## Abstract 2

Simultaneous electrophysiological recordings from CA1 of the rat hippocampus and medial prefrontal cortex reveal enhanced correlated activity between the two structures selectively during behavior that recruits spatial working memory.

## Introduction

The coordinated, rhythmic activity of neuronal populations gives rise to oscillations in local field potentials (LFP) and electroencephalograms at a broad range of frequencies [1]. Throughout the brain, these oscillations potentially constitute clocking mechanisms against which to reference and coordinate the timing of neural firing. Synchronization of these rhythmic activities is likely to reflect or underlie functional interactions between neurons within a defined brain structure, or between disparate populations in distinct structures [2,3]. Equally, abnormal synchronization may impair functional interactions and contribute to complex cognitive disorders such as schizophrenia [4,5] and attention deficit/hyperactivity disorder [6–8].

At the level of single neurons, synchrony is evident in the consistent temporal relationships between the firing patterns of interconnected cells. These are most commonly quantified using cross-correlation techniques [9–11]. For example, the correlated firing of cortical neurons is implicated in visual processing [12], attention [13], and motor learning [14]. However, the majority of these studies are based on recordings from single brain regions, and are unable to address the nature of correlated activities in networks spanning multiple structures. Do correlations also underlie coordination between anatomically and functionally related brain regions? If so, are they also mediated or reflected by oscillatory population activities at the LFP level?

Theta rhythms are 4- to 12-Hz oscillations consistently associated with complex behaviors presumed to require mnemonic processing and/or decision-making, for example spatial exploration in rodents [15], working memory in primates [16], and navigation and working memory in humans [17,18]. Dynamic, behavioral modulation of theta rhythms may therefore indicate or mediate cross-neuronal and/or cross-structural interactions during these behaviors. Theta rhythms are found in many mammalian brain structures, but are most prominent in the rodent hippocampus [19]. Here, the firing of individual “place cells”—hippocampal principal excitatory neurons with spatial receptive fields [20]—is coordinated (“phase-locked”) with respect to the local theta rhythm. Thus the action potentials of a given neuron tend to occur during a preferred phase of the theta cycle. This phase-locking of hippocampal spike-timing to ongoing LFP oscillations is an important example of temporal coding in the brain [21] and—in concert with the related phenomenon of phase precession—has been proposed to allow higher-order coding of spatial information than that imparted by the firing-rate pattern alone [22]. It has also been proposed that hippocampal theta rhythms may coordinate neural activity during sensorimotor integration [23] or information encoding [24,25].

Neuronal firing phase-locked to the hippocampal theta rhythm has also been described in cingulate cortex [26], amygdala [27], entorhinal cortex [28], striatum [29], and, most recently, the rat prefrontal cortex [30,31]. As in the hippocampus, phase-locking in the prefrontal cortex is also accompanied by phase precession [32]. This raises the possibility that phase-locking may play a broader role in defining the temporal relationships between cross-structural activities. However, it remains to be established how these phase relationships influence the firing of connected neurons, and how—or whether—they relate to behavior or hippocampal function. For example, while Siapas et al. [30] included radial arm maze and T-maze tasks among the conditions during which they collected data, analyses made no attempt to relate medial prefrontal cortex (mPFC) phase-locking to ongoing behaviors. If mPFC phase-locking remains constant while behavioral demands vary, it is unlikely to reflect functional interactions between coactive structures. Similarly, Hyman et al. [31] recorded during running on a linear track and random foraging, and were therefore unable to explicitly relate variations in phase-locking with variations in behavioral demands.

Siapas et al. [30] suggested that mPFC phase-locking may play some role in the formation of long-term memories that require that transfer of information from the hippocampus to the neocortex. Consistent with this role, monosynaptic projections from hippocampus to the deep layers of mPFC [33,34] do exhibit activity-induced plasticity [35], and LFP oscillations in the two structures are correlated during slow-wave sleep [36]. However, lesions or disruptions of either mPFC or the hippocampus impair spatial working memory [37–39], providing functional evidence that the two structures also interact on-line during behaviors that require short-term mnemonic processing [40]. To investigate the nature of functional interactions that relate to decision-making, we made simultaneous tetrode recordings of extracellular action potentials and LFP from CA1 and mPFC to examine coordination of activity in these two structures during a spatial working-memory task designed to dissociate neural activity related to performance (e.g., running and orienting behaviors) from activity related to mnemonic or decision-making processes. We find that spike timing and theta-rhythmic activities in CA1 and mPFC become more coordinated during epochs of the task associated with peak mnemonic and decision-making load.

## Results

### Behavior

**Figure 1:**
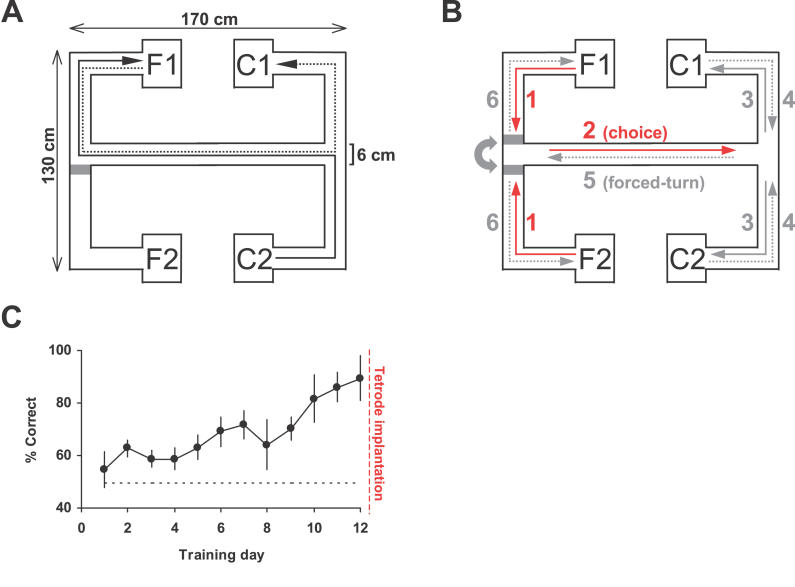
Experimental Design and Performance during the Spatial Working-Memory Task (A) Schematic of the maze and a pair of runs comprising a single trial (forced-turn direction C2 to F1, solid arrow; choice direction F1 to C1, dotted arrow). Grey rectangle marks the moveable barrier. (B) The task was broken down into distinct behavioral stages for analysis: 1, running away from the “cue” reward point towards the central arm; 2, crossing the central arm in the choice direction (only activity on the central three-quarters section of the arm was considered for analysis to avoid divergent routes near the turning points and inconsistent running behavior); 3, post-choice running to reward point (rats were rewarded for choosing C1 if the trial started at F1 and for choosing C2 if the trial started at F2); 4, returning to the central arm (where a second barrier blocked the route to the opposite reward point); 5, crossing the central arm in the “forced-turn” direction; and 6, returning to one of the two reward points (F1 or F2 chosen at random for each trial). Stages 1 and 2, marked by the red arrows, were presumed to involve working memory and/or decision-making. (C) The six rats were trained to asymptotic working-memory performance for at least 12 d before tetrode implantation.

Data are presented from eight recording sessions from six rats. Rats ran 15–25 trials per 20- to 30-min recording session. Each trial of the task comprised a “forced-turn” (sample) and “choice” (test) epoch (Figure 1A), and was subdivided into a number of stages corresponding to different sections of the maze (Figure 1B). Rats were trained to asymptotic performance prior to electrode implantation (Figure 1C), and performed the task at 83% ± 5.0% (mean ± standard error of the mean) correct during the eight sessions presented here. Rats began at a reward point (F1 or F2), then ran towards the central arm of the maze (stage 1 in Figure 1B). A moveable barrier directed them down the central arm towards the choice point (stage 2), when they were required to choose a left turn if the trial started from F1, or a right turn if the trial started from F2. If the correct turn was made (stage 3), chocolate reward was delivered remotely to C1 or C2; incorrect turns were not reinforced. Rats then returned to the choice point (stage 4) and were directed by another moveable barrier back down the central arm towards the forced-turn end of the maze (stage 5). Here, the barrier directed them to reward at F1 or F2 (stage 6), with left or right turns selected at random from trial to trial. The location of the forced-turn end of the maze was varied between animals.

Stages 1 and 2 (choice epoch) presumably invoked spatial working-memory processes: rats were required to “hold in mind” the location of the starting reward—or the direction of the turn between stages 1 and 2—in order to choose between C1 and C2 at the opposite end of the maze. By stage 3, their decision had already been made, and any working-memory requirement was negated. Stages 4–6 (forced-turn epoch) never required active working memory, since routes back to F1 or F2 were always predetermined by the barriers.

A primary concern during subsequent analyses was to dissociate overt behavioral differences (such as running speed) from differences in neural activity. For example, stages 1 and 6 both occurred on the same sections of the maze and yet made contrasting demands upon spatial working memory. However, rats ran more than twice as fast during stage 6 (44 ± 1.0 cm/s) than they did during stage 1 (18 ± 5.8 cm/s), a behavioral difference that may confound interpretation of differences between neural activities during the two stages. Similarly, rats ran at 17 ± 2.1 cm/s during stage 3 and at 31 ± 1.8 cm/s during stage 4. Most analyses therefore focused on the central three-quarters section of the central arm, where mean running speeds were similar during both choice and forced-turn epochs (38 ± 1.9 and 40 ± 2.0 cm/s, respectively). Restricting analyses to the central arm also excluded sections of divergent running trajectories at the very ends of the arm (Figure S1).

As rats crossed the central arm during forced-turn epochs, their route was predetermined by the moveable barrier. In contrast, during choice-direction epochs, rats were required to choose between left or right reward arms, employing spatial working memory to guide their decision. Since overt behavior was similar during both epochs, differences between neural activity during runs across the central arm in the forced-turn and choice directions therefore reflect mnemonic and/or decision-making processes, rather than the simple behavioral demands of the task.

### Behavioral Correlates of CA1 and mPFC Firing

One hundred and sixty-five mPFC and 149 CA1 neurons (ten neurons from ventral CA1) active on the maze were classified as putative pyramidal neurons or fast-spiking interneurons (4% of recorded mPFC neurons, 5% of CA1 neurons) on the basis of spike width, firing rate, and burst-firing characteristics. The classification was based on similar schemes derived from intracellular recordings [41,42] (Table 1), although only pyramidal neurons were used for subsequent analyses. The data of Siapas et al. [30] and Hyman et al. [31] set a precedent for examining the coordination of dorsal CA1 and mPFC activities. However, the most prominent hippocampal projections to mPFC arise from ventral CA1/subiculum [43]; initial experiments were therefore designed to compare the properties and interactions of dorsal CA1 neurons with ventral CA1/subiculum neurons. The low yield of well-isolated units from ventral regions precluded a systematic comparison between dorsal and ventral subregions in this study, although the basic properties of ventral CA1 pyramidal cells were comparable with those of dorsal neurons (see Table 1).

**Table 1:**
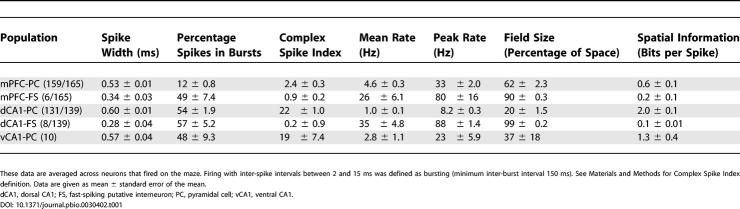
Spike Waveform, Burst Firing, Firing Rate, and Spatial Firing Parameters for the mPFC and CA1 Populations

**Figure 2:**
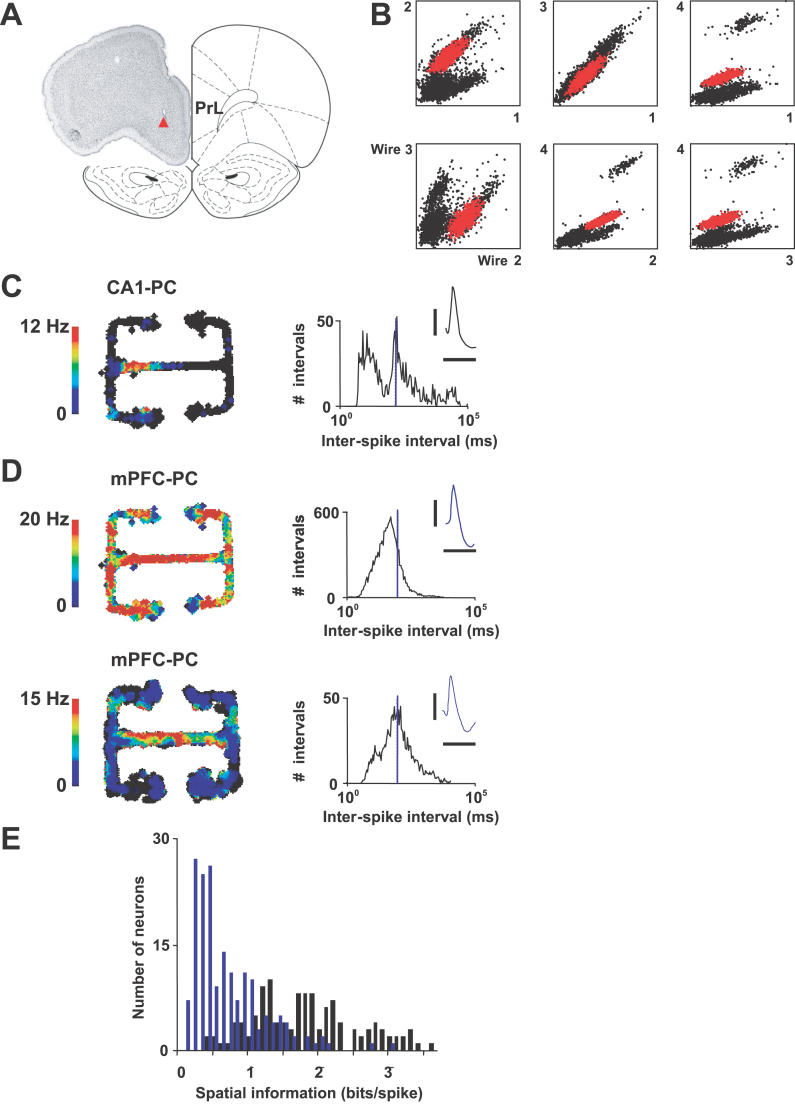
Recording Details and Typical Hippocampal and Prefrontal Firing Properties on the Maze (A) mPFC tetrodes targeted deep layers of prelimbic and infralimbic cortices. Photograph shows a typical lesion site (triangle) marking the tip of a tetrode. The partial brain section is superimposed on a schematic of a coronal section taken 3.7 mm rostral of bregma, showing the boundary of the prelimbic cortex (denoted by PrL). (B) Spike amplitude clusters for a typical mPFC tetrode. The cluster in red was for the neuron shown in D. The points in the six panels plot extracellular-action-potential amplitude on wire 1 of the tetrode versus wire 2, wire 1 versus wire 3, etc. (C) and (D) Activities of a typical CA1 place cell and two mPFC pyramidal cells, respectively (see also Figure S2). The upper mPFC neuron in D was recorded simultaneously with the CA1 neuron in C. Spikes were binned into positional pixels, and mean pixel firing rate was color-coded to generate the firing-rate maps on the left. Graphs show corresponding inter-spike interval distributions (10 Hz marked by the blue line; note logarithmic time scale). Waveforms show averaged extracellular action potentials recorded on a single wire of each tetrode (horizontal and vertical scale bars, 1 ms and 400 μV, respectively). (E) Overlap in the distributions of spatial information carried by spikes from CA1 (black) and mPFC (blue) populations.

The behavioral correlates of mPFC neuronal activity tended to be more spatially distributed than for CA1 neurons (Figure 2), with no population bias towards one maze region (mean mPFC firing rate 4.7 ± 0.4 Hz on reward arms [including reward points] and 5.2 ± 0.5 Hz on the central arm). Neither was mPFC population activity biased towards one task epoch. For example, overall central-arm firing rates were comparable in forced-turn and choice directions (5.3 ± 0.5 Hz and 5.2 ± 0.5 Hz, respectively).

**Figure 3:**
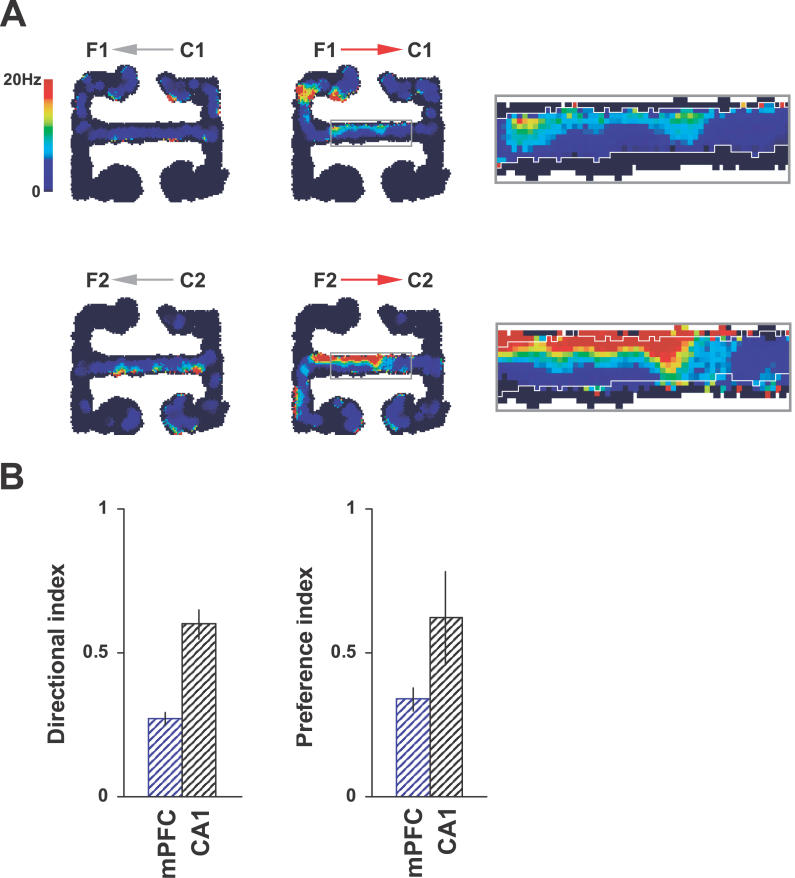
Directional Bias of mPFC and CA1 Firing Rates (A) Firing rate of a single mPFC neuron split into four trial types (shown by arrows). This neuron tended to fire at higher rates during runs in the choice direction, with the central arm firing highest during F2 → C2 trials. The magnified boxes show the central three-quarters section of the central arm. White lines mark the boundary of the positional pixels traversed by the rat on F1 → C1 trials. These are superimposed on the firing-rate map for F2 → C2 trials, showing the overlap between positions visited on both trial types. (B) Central-arm firing rates of both mPFC (blue) and CA1 (black) neurons tended to distinguish between runs in the forced-turn and choice directions (directional index > 0) and choice-direction runs in either F1 → C1 or F2 → C2 trials (preference index > 0; see Results). However, there was no overall tendency for CA1 or mPFC populations to fire at higher rates during any one epoch or trial type.

Despite these similar population firing rates during different task epochs, the central-arm firing rates of individual mPFC neurons did tend to distinguish between runs in the two directions and between different routes in the choice direction (Figure 3). A “directional index” for each neuron was defined as the magnitude of the difference between mean firing rates during forced-turn and choice directions, divided by the overall mean firing rate on the central arm. “Preference index” was defined as the magnitude of the difference in mean firing rates during F1 → C1 and F2 → C2 trials divided by the mean choice-direction firing rate. Thus both indices ranged from zero (firing rate identical in both epochs/routes) to one (fired only in one epoch). The mean directional index of mPFC neurons was 0.27 ± 0.02, and the mean preference index was 0.34 ± 0.04. This biased activity was also evident in CA1 (mean directional index 0.60 ± 0.05, preference index 0.62 ± 0.16) and was reminiscent of the CA1 activity previously described on similar linear tracks [44,45]. Slight differences in running trajectory or head direction may contribute to these firing-rate effects. However, these behavioral parameters did not vary consistently with trial type (Figure S1) and are unlikely to explain the firing-rate biases in their entirety. mPFC firing tended to be more sustained than that of CA1, with the average mPFC neuron firing at more than 10% of its maximum rate across 52% ± 3% of the central-arm area, whereas average CA1 place-cell firing covered only 22% ± 2% of the central arm. Similar “delay firing” activity in rat mPFC has been reported by Baeg et al. [46]. However, these firing-rate data do not address the nature of interactions between CA1 and mPFC.

### Behavioral Modulation of Cross-Correlations between CA1 and mPFC Spike Times

We first investigated coordination of hippocampal and prefrontal activities by quantifying the temporal alignment of neuronal firing using cross-correlation of spike times from CA1–mPFC unit pairs coactive during different behavioral epochs. Peak cross-correlation coefficients (bin size 100 ms, maximum lag ± 200 ms) were normalized by spike counts, and bias-corrected by subtracting values obtained when trials were shuffled with respect to one another. Spike trains from 50 CA1–mPFC pairs coactive during stages 1 and 6 (see Figure 1B) showed significantly higher cross-correlation coefficients during stage 1 than during stage 6 (0.029 ± 0.002 versus 0.017 ± 0.002; *p* < 0.01 by Wilcoxon rank sum test on animal means). These differences cannot be explained by changes in firing rate, as the mean firing rates of these neurons were not significantly different during the two epochs (6.3 ± 2.1 Hz and 5.9 ± 2.0 Hz for epochs 1 and 6, respectively, in CA1; 7.7 ± 1.1 Hz and 8.8 ± 1.7 Hz in mPFC). However, running speeds were lower during stage 1 than during stage 6 (see Behavior); could this explain the differences in correlated activity? To address this, we also compared the degree of correlation between 49 coactive CA1–mPFC unit pairs during stages 3 and 4, neither of which required spatial working memory. Again, running speeds were higher during stage 3 than during stage 4. However, mean cross-correlations were not significantly different under these conditions (0.019 ± 0.002 during both stages). Independent of spatial location (epochs 1 and 6 correspond to the same sections of the maze), correlations between CA1 and mPFC activities were therefore significantly enhanced during the epoch presumed to recruit spatial working memory. These data are summarized in Table 2.

**Table 2:**
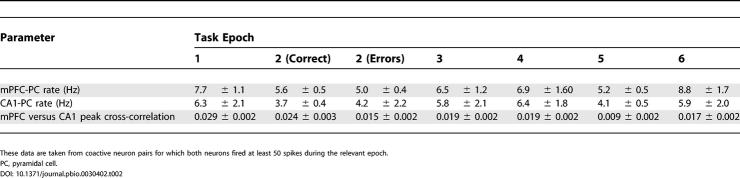
Summary of Mean Firing Rates and Mean Peak Cross-Correlation Coefficients between CA1–mPFC Neuron Pairs during the Different Task Epochs Shown in Figure 1B

**Figure 4:**
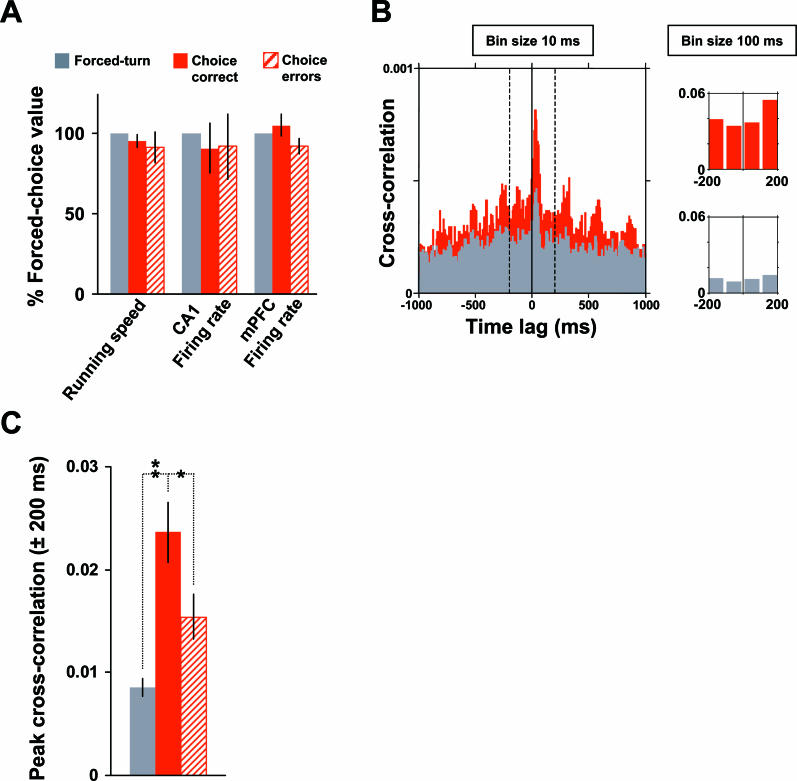
Enhanced Cross-Correlations between Spike Trains of CA1–mPFC Neuron Pairs during Behavioral Epochs Requiring Working Memory and Decision-Making (A) Mean running speeds, CA1 firing rates, and mPFC firing rates were comparable during forced-turn (grey, epoch 5), choice-correct (red, epoch 2), and choice-error runs (hatched red) across the central arm. (B) Example cross-correlogram (bin size 10 ms, maximum time lag ± 1,000 ms) for a single CA1–mPFC neuron pair (referenced to the CA1 firing at time 0), showing that correlated activity was higher during choice runs (red) than forced-turn runs (grey). The width of the central peak at 50% of its maximum value is 120 ms. This compares with a mean peak width of 156 ± 41 ms for 29 neuron pairs with peak cross-correlation coefficients (bin size 10 ms) of at least 0.0005. For comparison across task epochs, peak cross-correlation coefficients were quantified at the ± 200-ms time range with a bin size of 100 ms (inset to the right). (C) Mean correlations for all neuron pairs that fired at least 50 spikes each during the three run types. CA1–mPFC correlation coefficients were significantly higher during choice runs (72 pairs) than during forced-turn (72) or error runs (49 pairs; ** *p* < 0.01, * *p* < 0.05 Wilcoxon rank sum test for grouped animal means).

In order to confirm that differences in correlated activity related to varied working-memory or decision-making processes rather than overt behavioral state, we compared CA1–mPFC cross-correlations on the central arm during forced-turn and choice-direction epochs (stages 5 and 2), when running behavior was at its most uniform (Figure 4). Furthermore, we subdivided choice-direction runs into correct and incorrect trials. For 72 unit pairs coactive on the central arm, the mean peak cross-correlation during forced-turn runs was 0.009 ± 0.002 (Figure 4C). Correlated activity was significantly higher during choice-direction runs on correct trials (0.024 ± 0.003; *p* < 0.01). Importantly, 49 CA1–mPFC neuron pairs coactive during error trials showed significantly reduced correlations relative to correct-choice trials (0.015 ± 0.002; *p* < 0.05).

These data demonstrate that cross-structural synchronization of neuronal firing at the neuron-pair level is modulated during this working-memory task. Specifically, mPFC activity is more highly correlated with CA1 place-cell activity during epochs of the task associated with spatial working-memory or decision-making processes. This synchronization may reflect the transfer of hippocampal spatial information to a mPFC working-memory system. Consistent with this, mPFC firing during runs in the choice direction carried significantly more spatial information [47] than during forced-turn runs (0.30 ± 0.06 versus 0.24 ± 0.05 bits per spike, two-tailed *p* < 0.05, Student's *t*-test). Rats may make errors in this task for a number of reasons, including failures of attention, working memory, or decision-making. Nevertheless, the attenuated cross-correlation of CA1–mPFC activity on the central arm during error trials does indicate that coordinated hippocampal–prefrontal activity is selectively associated with accurate behavioral performance.

### Phase-Locking of CA1 and mPFC Spike Times to Local and Remote Theta Rhythms

How might the level of cross-structural coordination evident in the cross-correlation analysis be orchestrated and modulated? Siapas et al. [30] recently described phase-locking of mPFC spike-timing to the CA1 theta rhythm. During periods of phase-locking, spikes tend to occur during consistent time windows imposed by ongoing theta rhythms. It follows that the relative timing of spikes from multiple phase-locked neurons may also become more consistent under these conditions. Indeed, Siapas et al. [30] showed that mPFC neurons significantly phase-locked to the CA1 theta rhythm showed greater covariance with CA1 spike-timing than non-phase-locked neurons. However, this previous study made no attempt to link phase-locking to function by examining its relationship to ongoing behavior. We therefore went on to examine phase-locking of mPFC neurons during different epochs of this spatial working-memory task.

**Figure 5:**
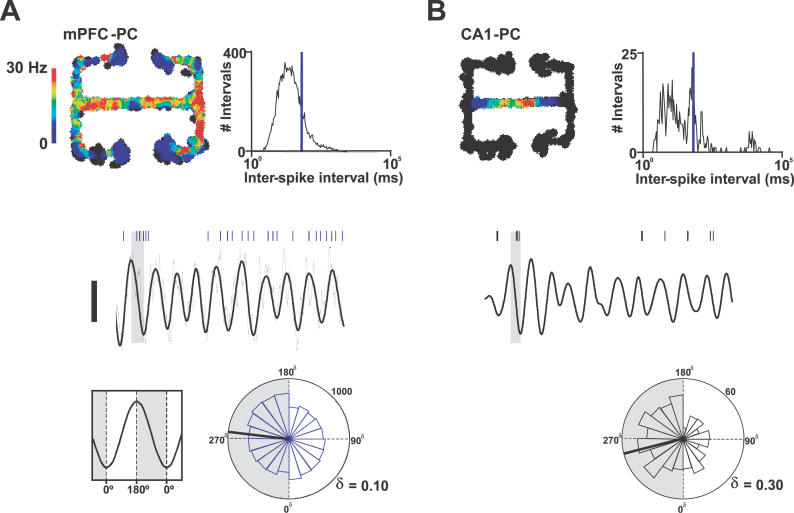
Spike-Timing in CA1 and mPFC Populations Was Phase-Locked to CA1 Theta Rhythm Firing-rate maps for representative mPFC (A) and CA1 (B) pyramidal cells. Graphs show inter-spike interval distributions (blue line marks 10 Hz). Thick black lines show CA1 LFP band pass filtered at 4–12 Hz during single central-arm crossings (scale bar 0.5 mV, length 1.3 s and 1.4 s in A and B, respectively) with spike times of the two neurons above marked by ticks. Raw LFP is shown by the thin black line in A. Rose histograms show phase distributions for these single mPFC (blue) and CA1 (black) neurons with respect to CA1 theta rhythm. Thick black lines mark mean preferred phase. The numbers on the outer circular axis give spike counts. Circular-concentration coefficients are given by κ. Both distributions are significantly nonuniform (*p* < 0.01, Rayleigh test).

As detailed by Siapas et al. [30], the degree of phase-locking can be quantified by the circular-concentration coefficient, κ, of each neuron's phase distribution. κ was estimated by the maximum-likelihood method [48] and is a measure of concentration around the mean preferred phase; κ is inversely related to the Rayleigh *p*-value, with κ = 0 for uniform distributions. Phase distributions of CA1 pyramidal-cell spikes with respect to the local CA1 theta rhythm were significantly nonuniform for 71% of the population (Rayleigh test of uniformity *p* < 0.01). The mean circular-concentration coefficient across the entire population (including neurons with statistically uniform phase distributions) was κ = 0.21 ± 0.01. Adding random jitter of up to 100 ms to the spike times of these cells reduced mean κ to 0.05 ± 0.02, close to the zero value expected for uniform distributions. As expected, a cross-structural phase relationship was also evident: mPFC firing was significantly phase-locked to CA1 theta rhythm in 49% of the population (mean κ = 0.08 ± 0.01 for the entire population averaged over all firing on the maze; see examples in Figure 5). Again, adding random jitter to mPFC spike times reduced circular-concentration coefficients to 0.04 ± 0.02.

Siapas et al. [30] suggested that CA1 activity tends to lead to mPFC activity, as mPFC firing locked more reliably to the preceding CA1 theta cycle than to the simultaneous oscillation. In agreement with this, we found that shifting the relative timing of CA1 LFP forward by an average of 30 ± 10 ms maximized the values of κ for mPFC phase distributions. In contrast, shifting the timing of CA1 theta relative to CA1 spike times (by times of up to ± 100 ms) did not significantly improve their phase relationship.

The rhythmic activity underlying hippocampal theta is particularly apparent in LFP recordings because of the laminar organization of current sinks and sources in CA1. The nature of neocortical LFP is more ambiguous, yet we were able to distinguish clear instances of theta-frequency oscillations in mPFC LFP during running on the maze. These were distinct from the 7- to 12-Hz high-voltage spindles observed during immobile states [49,50] (data not shown). We therefore also calculated phase distributions for the firing of each neuron in relation to mPFC theta peak times. Significantly nonuniform distributions (κ = 0.05 ± 0.01 and κ = 0.09 ± 0.01) were shown by 29% of CA1 and 43% of mPFC populations, respectively. Importantly, the circular-concentration coefficient of any given neuron versus CA1 theta was positively correlated with its circular-concentration coefficient versus mPFC theta (*r* = 0.65, *p* < 0.001), indicating that theta-frequency activities in the two structures were related.

### Behavioral Modulation of Phase-Locking

If the consistent timing relationship between neural spiking and ongoing theta rhythms provides a mechanism through which to coordinate mPFC and CA1 activity, and if the coordination between these structures depends upon task demands, the degree of phase-locking might be expected to vary with task epoch. In particular, phase-locking should be enhanced during the task epochs associated with working memory or decision-making and enhanced CA1–mPFC spike cross-correlations.

**Figure 6:**
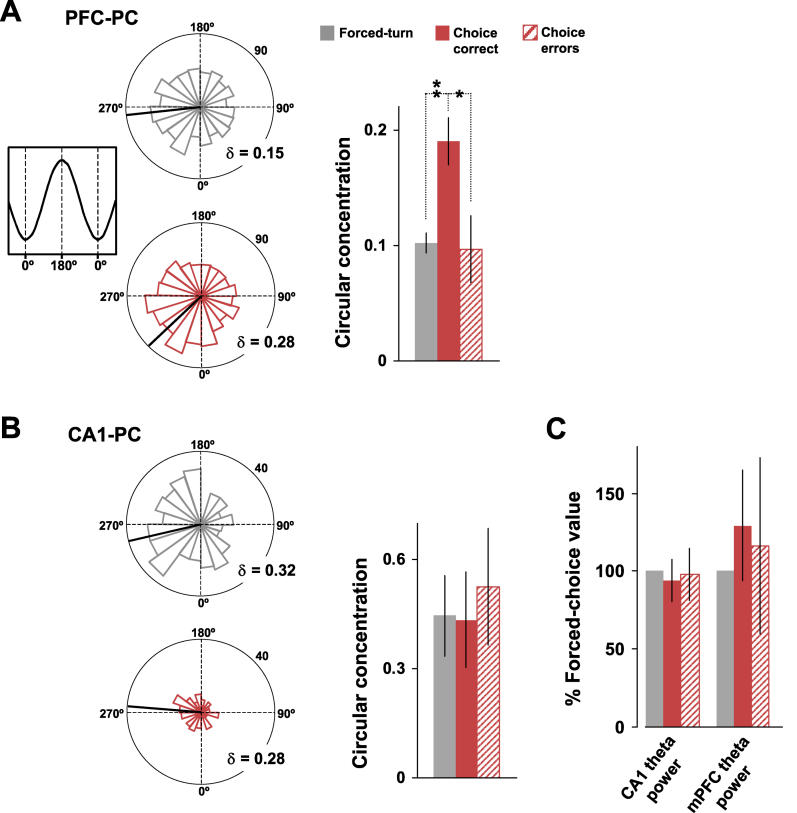
Theta Phase-Locking of mPFC Spike Timing to the CA1 Theta Rhythm Was Enhanced during Choice Epochs Relative to Forced-Turn and Choice-Error Runs Phase distributions for single mPFC (A) and CA1 (B) neurons during forced-turn (grey), choice-direction (red), and choice-error (hatched red) epochs. Bar graphs show mean-population circular-concentration coefficients (κ) during the three epochs, and the significant (** *p* < 0.01, * *p* < 0.05) increase in κ for the mPFC population during choice epochs (39 neurons) relative to forced-turn and choice-error epochs (39 and 27 neurons, respectively). In contrast, the CA1 population showed a similar degree of phase-locking during all epoch types (for 26, 26, and 15 neurons, respectively). (C) The results in A cannot be explained by changes in mean LFP theta power, which was comparable during forced-turn, choice, and choice-error epochs in both CA1 and mPFC.

In every animal tested, the spikes of mPFC units active on the central arm showed a greater concentration around their preferred phase of CA1 theta during choice-direction epochs on correct trials than during forced-turn epochs (Figure 6A). Thus, although approximately 40% of the active mPFC population showed significantly uniform phase distributions in both epochs (44% during choice and 39% during forced-turn), the circular-concentration coefficients of these distributions were significantly higher during correct, choice-direction trials (κ = 0.19 ± 0.02 versus κ = 0.10 ± 0.02, *n* = 39; *p* < 0.01 Wilcoxon rank sum test based on animal means). Furthermore, as for the spike train cross-correlations, phase-locking of mPFC neurons on the central arm was significantly attenuated during error trials (κ = 0.10 ± 0.03, *n* = 27; *p* < 0.05 versus correct trials). In contrast, the degree of phase-locking shown by CA1 neurons was similar during choice-correct, forced-turn, and choice-error epochs (Figure 6B; κ = 0.43 ± 0.13, *n* = 16; κ = 0.45 ± 0.11, *n* = 16; κ = 0.53 ± 0.16, *n* = 10, respectively).

The increases in phase-locking of mPFC neurons were not accompanied by overt changes in running behavior, increased population firing rates (see Figure 4A), or increased theta power (see Figure 6C) during choice epochs. They must therefore reflect a more consistent timing relationship between mPFC spikes and the CA1 theta rhythm during the behavioral epochs associated with peak working-memory load and decision-making. Again, error trials were associated with impaired coordination between CA1 and mPFC activities. Thus enhanced phase-locking of mPFC activity to the CA1 theta rhythm paralleled the increases in correlated activity at the neuron-pair level, consistent with the suggestion that phase-locking to the theta rhythm constitutes a mechanism through which to temporally coordinate populations of neurons in these two structures.

### Coherence between CA1 and mPFC LFP

**Figure 7:**
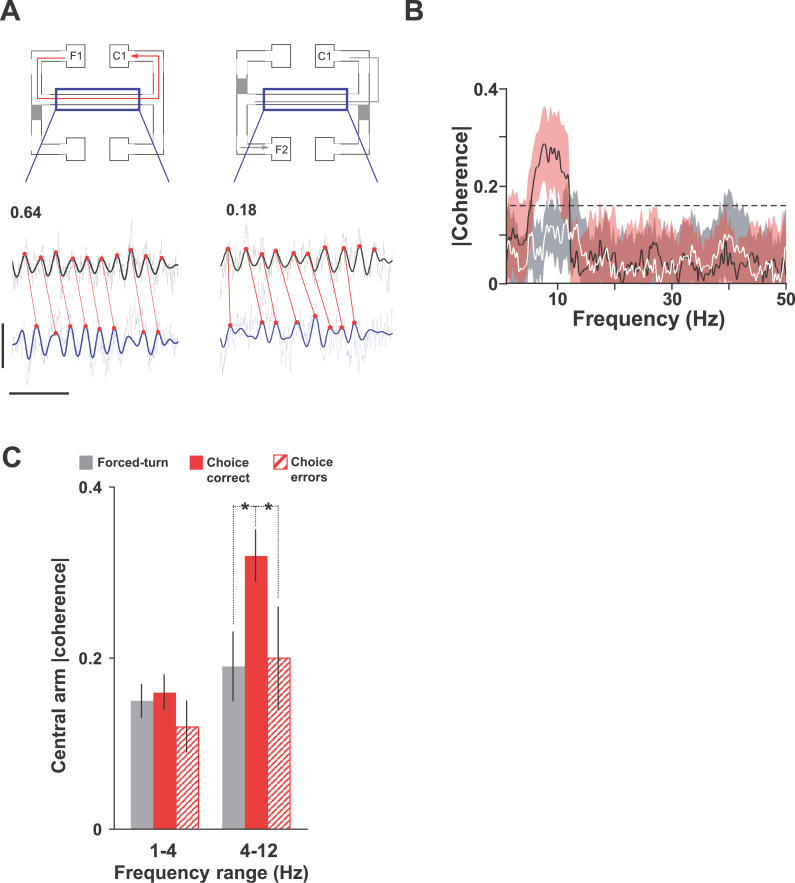
CA1–mPFC LFP Coherence Showed a Significant Peak in the Theta-Frequency Range and Was Enhanced during Choice Epochs (A) Raw LFP from dorsal CA1 (black) and mPFC (blue) during consecutive single central-arm crossings in the choice (left) and forced-turn (right) directions. Thick lines show theta-filtered LFP. Horizontal scale bar 0.5 s; vertical scale bar 0.8 mV. Red lines highlight the timing relationship between CA1 and mPFC theta peaks (red circles). Numbers above raw LFP traces give coherence in the 4- to 12-Hz range during these two example trials. (B) Trial-averaged, central-arm coherence during a single run-session (17 trials). Central-arm coherence is subdivided into forced-turn (grey) and choice (red) directions. Dashed line marks 95% confidence level, with shaded band thickness corresponding to jackknife error bars (estimated over trials and nine tapers). Significant coherence was seen only in the theta-frequency range, and only during choice epochs on the central arm. (C) Mean coherence at delta (1–4 Hz) and theta (4–12 Hz) frequencies, pooled across animals during forced-turn (grey), choice-correct (red), and choice-error (hatched red) epochs (* *p* < 0.05). Like theta CA1–mPFC spike cross-correlations and theta phase-locking of mPFC units, theta-frequency CA1–mPFC LFP coherence peaked during choice-direction runs across the central arm.

The enhanced phase-locking of mPFC single-unit activity to the theta rhythm suggests a broader coordination of CA1 and mPFC population activities in the theta-frequency band. A direct measure of such covariation at the population level is the coherence between LFPs in the two structures. LFP measures of “averaged” population activity constitute a useful adjunct to the spike-timing analyses, since they are unlikely to be sensitive to trial-by-trial fluctuations in the firing rates of individual neurons. We quantified multi-taper estimates of coherence in the same 4- to 12-Hz theta-frequency range as unit-LFP phase-locking (Figure 7). Considering only LFP sections corresponding to central-arm crossings, theta-frequency coherence assessed on a trial-by-trial basis was significant during 55% ± 2% of crossings in the choice direction, and only 32% ± 6% of crossings in the forced-turn direction. Hence the absolute value of mean 4- to 12-Hz coherence on the central arm was significantly higher during choice epochs on correct trials than during forced-turn epochs (0.32 ± 0.03 versus 0.19 ± 0.04, respectively; *p* < 0.05). Like the cross-correlation and phase-locking measures, this measure of CA1–mPFC coordination was reduced during error trials (to 0.20 ± 0.06, *p* < 0.05, versus correct trials).

Given the restricted sample lengths (2–2.5 s for central-arm crossings) and 4-Hz bandwidth, coherence estimates at frequencies below 4 Hz are not statistically robust. Nevertheless, mean 1–4 Hz (delta) coherence did not differ markedly between choice-correct, forced-turn, and choice-error epochs (0.16 ± 0.02, 0.15 ± 0.02, and 0.12 ± 0.03, respectively). Neither did we find any consistent coherence at frequencies above 12 Hz. LFP coherence therefore paralleled phase-locking of mPFC units to the CA1 LFP, both in terms of its theta-frequency range and its enhancement during behavioral epochs that required spatial working memory or decision-making.

## Discussion

A critical role of working memory is the dynamic and selective incorporation of task-relevant information into decision-making processes [51]. Working memory therefore exemplifies conditions during which multiple disparate brain structures must interact transiently yet coherently. The hub of these interactions is presumed to lie in the prefrontal cortex, whose working-memory functions subserve its broader, integrative roles in establishing context and guiding behavior appropriately [52]. Within the cortex, there is mounting theoretical [53] and electrophysiological evidence from humans and primates [16–18] suggesting a role for rhythmic activity in working memory. Do cortical rhythms relate to rhythms elsewhere in the brain? Are they indicative of a broader functional network, allowing subcortical structures to participate in decision-making processes? How are oscillations recorded at the LFP level reflected by the activities of single neurons? Which frequency bands are key, and do different frequencies play different roles?

Our simultaneous recordings from prefrontal cortex and hippocampus during spatial working memory provide evidence for the rapid configuration of functional connectivity through the theta-frequency entrainment of oscillatory networks across these two brain regions. This entrainment was specific to a 4- to 12-Hz frequency range and was evident at every level examined, from individual pairs of coactive neurons, to the theta phase-locking of neurons to LFP, to hippocampal–prefrontal LFP coherence.

The behavioral correlates of mPFC firing during this task were varied; mPFC activity presumably reflects and drives many aspects of behavior. However, the firing of a significant proportion of the mPFC population did carry spatial information in the hippocampal range. In particular, increased spatial information content of mPFC firing coincided with epochs of increased phase-locking to hippocampal theta. Therefore, rather than mPFC neurons “becoming place cells” during this spatial task, the selective refinement of phase-locking between mPFC pyramidal-cell firing and ongoing theta rhythms acted alongside enhanced theta-frequency coherence to integrate hippocampal and prefrontal activities when required by the task.

Behaviorally modulated phase-locking potentially imparts great flexibility to the mPFC, allowing any given mPFC neuron to join different functional networks according to prevailing behavioral demands and in line with its current relationship to ongoing hippocampal activity. The periodicity of 4- to 12-Hz theta rhythms may make them particularly suitable reference signals. For example, groups of mPFC and CA1 neurons locked to the downward (>180°) phase of theta will have mutually increased firing probabilities during repeated ∼50-ms windows. The net effect will be increased correlated activity amongst these groups of neurons. This proved to be the case during choice epochs, when improved cross-correlations between CA1–mPFC unit pairs paralleled enhanced phase-locking and coherence. Similarly, the value of each neuron's mean preferred phase during a given behavioral epoch will consistently dictate the order in which that neuron fires relative to neurons with different mean preferred phases. Theta rhythms can therefore mediate the consistent timing relationships needed to establish functional connectivity between two structures, in this case serving to dynamically incorporate currently relevant spatial information into decision-making processes.

How is CA1–mPFC synchrony enhanced during specific behavioral epochs? One possibility is simply through simultaneously increased theta-modulated activity in these two structures. However, the enhanced correlations and phase-locking were independent of changes in theta power, since overall theta power was similar during forced-turn and choice epochs. In addition, the coherence measures are normalized by the power spectra of CA1 and mPFC LFPs and are consequently insensitive to power changes. Together, these data therefore imply selective alignment of CA1 and mPFC theta-rhythmic activity during choice-direction runs across the central arm of the maze. Since the instantaneous frequency of theta rhythms can vary rapidly over time, this alignment requires active synchronization of CA1 and mPFC rhythms in order to maintain their coordination with respect to each other. Similarly, maintenance of enhanced phase-locking of mPFC neurons to hippocampal theta cannot arise simply as a consequence of firing rates and LFP being modulated similar frequencies, but rather necessitates precise temporal control of spiking relative to theta rhythms [30].

This level of control may be exerted by monosynaptic projections from the hippocampus, which have direct influence on mPFC interneurons [54]. Since the synchronization of oscillatory networks is often attributed to the activity of local inhibitory interneuronal networks [55], these projections may provide the anatomical and physiological foundations for coherence between these two structures. Interestingly, the most dorsal part of CA1 does not contribute direct projections to mPFC [43], raising the possibility that dorsal CA1 interacts with mPFC via ventral CA1/subiculum. Whilst we did not observe qualitative differences between ventral and dorsal CA1 neurons in terms of their correlations with coactive mPFC neurons or their phase-locking properties, further experiments are required to establish the functional consequences of the known anatomical connections in this system.

There is no evidence for direct reciprocal projections from mPFC back to the hippocampal formation [56], making it tempting to speculate that the hippocampus drives mPFC firing rather than vice versa. Although our data do not unequivocally address the directionality of hippocampal–prefrontal interactions, this is supported by Siapas et al. [30], who suggest that mPFC neurons phase-lock to CA1 theta that occurs ∼50 ms in advance of their spikes; this also proved to be the case during the working-memory task employed here. However, whether behavioral-dependent enhancement of these coordinated activities is achieved entirely through hippocampal–prefrontal connectivity or via some third party that influences both CA1 and mPFC remains to be established.

Whilst enhanced theta-frequency coordination coincided with peak working-memory load, it is possible that theta-frequency interactions between CA1 and mPFC do not pertain solely to working memory or decision-making. For example, attention or reward expectancy may also vary between choice and forced-turn behavioral epochs. It should also be noted that some degree of phase-locking and coherence remained evident during runs across the central arm in the forced-turn direction. These residual interactions may reflect some working-memory-related aspect of spatial behavior common to both task epochs, such as updating route or task-rule information. CA1–mPFC synchrony—neuron-pair correlations, phase-locking, and LFP coherence—also fell to these control levels during runs towards the choice point on error trials. We cannot determine the stage of the task at which the errors originated, or whether they were due to failures in mnemonic, attentional, or decision-making components. However, the fact that the degree of CA1–mPFC synchrony can be used to predict behavioral outcome strongly suggests that these electrophysiological phenomena are indeed signatures of cross-structural interactions.

In summary, our data reveal correlations between behavioral demands and cross-structural neural synchrony: theta-frequency coordination between CA1 and mPFC peaks during behavioral epochs presumed to require effective communication between these two structures. It follows that disruption of such complex cross-structural communication is likely to generate behavioral impairments. For example, schizophrenia is associated with altered GABAergic function in hippocampal and prefrontal interneurons [57], and is widely presumed to involve disrupted functional connectivity of the prefrontal cortex [4,58,59]. Interestingly, schizophrenic patients do show spatial working-memory impairments [60]. The theta-rhythm-mediated coordination of hippocampal–prefrontal activity that we describe here may reflect the nature of cross-structural coordination at network and neuronal levels, and may contribute to both the clinical diagnosis of the impaired interactions likely to underlie cognitive disorders and to characterizing animal models of these diseases.

## Materials and Methods

All procedures were performed in accordance with the Massachusetts Institute of Technology Committee on Animal Care and the National Institutes of Health guidelines. Six male Long-Evans rats (2–6 mo) were mildly food-deprived (to 85% of free-feeding body weight) and trained to run a continuous spatial-alternation task (see Figure 1A). Each trial comprised distinct sample and test epochs. The contingency was set such that, for example, a rat forced to turn to his right during the forced-turn epoch had to choose a left-hand turn to win reward during the subsequent choice epoch. Forced-turn direction was varied randomly, with no more than three consecutive trials in one direction. The relative location of the forced-turn end of the maze was varied between animals. Every effort was made to constrain running to overlapping linear trajectories by using a narrow track (6 cm). Furthermore, analysis epochs excluded reward points and turning points.

Each rat was trained to asymptotic performance (two consecutive days of at least 80% choice-correct) over a period of 12–14 d before surgery, then implanted with arrays of adjustable tetrode recording electrodes targeted to the mPFC (+3.2 mm, +0.6 mm from bregma) and ipsilateral dorsal CA1 (−3.6 mm, +2.2 mm). In two rats, tetrodes were also targeted to ventral CA1 (−6.3 mm, +6.2 mm). Differential recordings of extracellular action potentials (sampled at 31.25 kHz per channel, filtered between 600 Hz and 6 kHz) and continuous LFP (sampled at 3.125 kHz per channel, filtered between 1 and 475 Hz) were made using Keithley Instruments acquisition boards (DAS-1802HC [http://www.keithley.com]). Local reference electrodes were placed in overlying (for dorsal CA1 recordings) or adjacent (for ventral CA1 recordings) white matter, or in a proximal cortical region without spiking activity (2.4–2.7 mm below the pial surface for mPFC recordings). Positioning in white matter was achieved on the basis of characteristically flat LFP recordings (with no hippocampal sharp waves or ripples) and an absence of action-potential activity in this region. Only hippocampal LFP data taken from dorsal CA1 are presented here. Electrolytic lesions established tetrode tip positions at the end of each experiment (Figure 1A).

Data presented here are taken from eight recording sessions from the six rats. Action potentials were assigned to individual neurons by off-line, manual clustering using Xclust software (M. A. Wilson). Subsequent analyses employed a combination of in-house software (M. A. Wilson) and custom Matlab code (MathWorks, Natick, Massachusetts, United States). Firing with inter-spike intervals of between 2 and 15 ms was defined as bursting (minimum inter-burst interval 150 ms). The Complex Spike Index (Table 1) combined a measure of bursting with a measure of the likelihood that spikes later in bursts were smaller in amplitude than spikes earlier in bursts [61]. Spatial information was calculated according to Skaggs et al. [47]. Most analyses compared firing on the central three-quarters section of the central arm in the two running directions, and were restricted to neurons that fired at least 50 spikes in both choice and forced-turn epochs (in order to allow reliable circular statistics).

LFPs were down-sampled (to 600 Hz) and band pass filtered between 4 and 12 Hz, then maxima and minima detected and thresholds established to extract theta peak and trough times. Only peaks or troughs greater than one standard deviation from the mean amplitude of the filtered LFP were included (63% ± 6.6% of all maxima during choice epochs, 61% ± 9.5% during forced-turn epochs). Each spike was assigned a theta phase between 0 and 360° by linear interpolation of the spike time relative to the enveloping pair of peak (phase 180°) and trough (phase 0 or 360°) times. The Rayleigh test of uniformity was used to assess the resulting phase distributions for deviations from the circular uniform distribution. Circular statistics were calculated according to Fisher [48].

Multi-taper spectral analysis [62] was used to calculate power spectra and coherence for LFP data. This technique takes advantage of short-time-window Fourier analysis to reduce artifacts caused by non-stationary elements in the data (since data can be assumed to be stationary within the short sliding time-windows). The significance of trial-by-trial magnitude of the coherence during central-arm crossings was calculated according to Jarvis and Mitra (with coherence values greater than 2/√[(number of trials) × (number of tapers)] considered significant at *p* = 0.05) [63]. Data in the text and figures are given as mean ± standard error of the mean. Statistical comparisons between forced-turn, choice-correct, and choice-error conditions were performed on the two groups of animal means using Wilcoxon rank sum tests.

## Supporting Information

Figure S1 Details of Movement Trajectories and Running Speed (A) Raw data showing positional samples (taken from LEDs mounted on the rat's head, 30-Hz sampling rate) during a single run-session. Blue box marks the central three-quarters section of the central arm used throughout analyses comparing choice and forced-turn directions. Scale bar 6 cm.
(B) Mean central-arm trajectories averaged across all rats and trials for the different behavioral epochs (± 1 standard deviation marked by the width of the shaded area). Upper panel compares choice-correct runs (solid red line and dark red shading) with choice-error runs (dashed red line and lighter shading). Lower panel compares choice-correct with forced-turn runs (grey line and shading). Trajectories show considerable overlap on this section of the maze; systematic variations in trajectory are therefore unlikely to explain the enhanced coordination seen during choice-direction runs.
(C) Mean running speeds across the central arm (same color scheme as in [B]).
(2.8 MB PDF).

Figure S2 Raster Plots Illustrating Trial-By-Trial Firing on the Central Arm of the Maze for the Three Neurons Shown in Figure 2
A CA1 pyramidal cell is shown in (A), while (B) and (C) show mPFC pyramidal cells; the two neurons in A and B were recorded simultaneously. Spikes (shown by the tick marks on the rasters) were parsed into choice-direction (middle column) and forced-turn-direction (right column) runs. Corresponding linearized mean firing rates (Gaussian-smoothed, with a kernel width of 8 cm) are shown below each raster.
(2.3 MB PDF).

## Abbreviations

LFP: local field potentials
mPFC: medial prefrontal cortex

## Abbreviations

LFP: local field potentials
mPFC: medial prefrontal cortex
