# Supplementary material for: Theta Rhythms Coordinate Hippocampal–Prefrontal Interactions in a Spatial Memory Task
Source: PLoS Biol. 2005 Nov 15;3(12):e402. doi: 10.1371/journal.pbio.0030402 (PMC1283536; doi:10.1371/journal.pbio.0030402)
Supplement: Supplementary file 1 [file pbio.0030402.sg001.pdf]

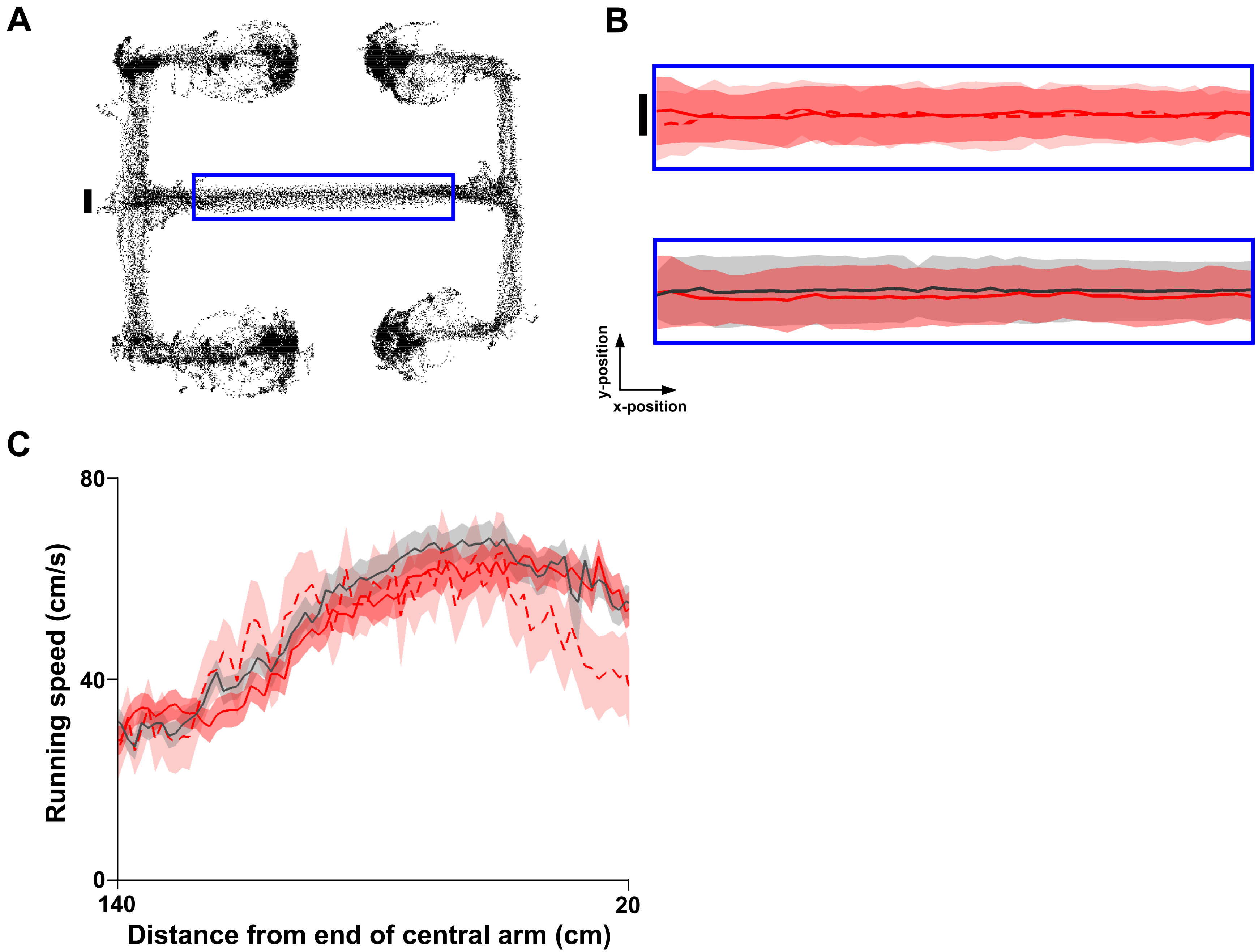

A
B
y-position
x-position
C
Running speed (cm/s)
80
40
0
140
20
Distance from end of central arm (cm)
