# Supplementary material for: Theta Rhythms Coordinate Hippocampal–Prefrontal Interactions in a Spatial Memory Task
Source: PLoS Biol. 2005 Nov 15;3(12):e402. doi: 10.1371/journal.pbio.0030402 (PMC1283536; doi:10.1371/journal.pbio.0030402)
Supplement: Supplementary file 2 [file pbio.0030402.sg002.pdf]

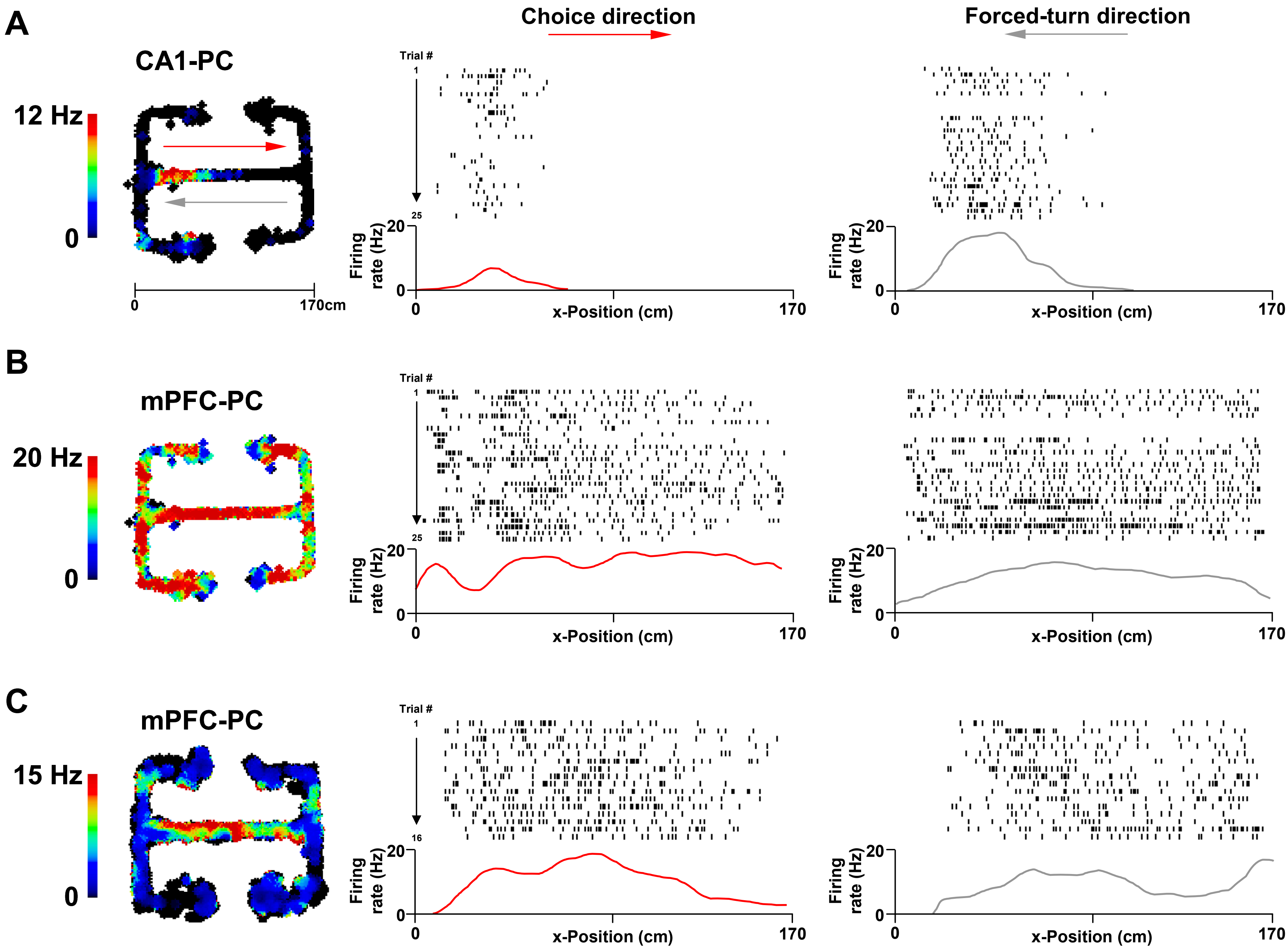

A
B
C
CA1-PC
mPFC-PC
mPFC-PC
12 Hz
20 Hz
15 Hz
0
0
170cm
Choice direction
Forced-turn direction
Trial #
1
25
16
Firing rate (Hz)
20
x-Position (cm)
170
